# Research Progress on the Nutritional Components, Bioactivity, Health Effects, and Food Applications of Passion Fruit Peel (PFP)

**DOI:** 10.3390/foods14193397

**Published:** 2025-09-30

**Authors:** Liangjie Ba, Chenglin Luo, Xue Li, Sen Cao, Donglan Luo

**Affiliations:** 1 School of Food Science and Engineering, Guiyang University, Guiyang 550005, China; baliangjie@163.com (L.B.); mw011206@163.com (C.L.); lixuez2025@163.com (X.L.); cs5638myself@126.com (S.C.); 2 College of Biological and Environmental Engineering, Guiyang University, Guiyang 550005, China

**Keywords:** application, bioactivity, nutritional information, food industry, passion fruit peel

## Abstract

Passion fruit peel (PFP) is a common byproduct of industrial passion fruit processing, yet it serves as a valuable source of diverse bioactive compounds and nutrients. However, limited attention has been paid in the literature to the nutritional properties and practical applications of PFP. This review summarizes methods for extracting bioactive substances from PFP, examines their potential health benefits, and explores their prospects for utilization in the food industry. Recent studies have quantified various bioactive components, such as flavonoids, vitamins, and dietary fiber (DF), while reporting the corresponding extraction yields or concentrations. Furthermore, these compounds exhibit significant potential in promoting human health, including antioxidant, anti-inflammatory, and gut health-improving effects. The analysis also highlights the bioavailability of bioactive constituents in PFP. Consequently, PFP presents a promising yet underexplored area for scientific research, though substantial challenges remain in optimizing its utilization, enhancing extraction efficiency, and advancing innovative applications.

## 1. Introduction

Passion fruit (*Passiflora edulis* Sims) belongs to the Passifloraceae family of the Passiflora genus and is widely distributed in tropical America, Asia and Africa. Generally, passion fruit can be classified into purple-skinned passion fruit and yellow-skinned passion fruit based on its appearance [1]. It is the third most popular tropical fruit in the world after mangoes and pineapples [2,3,4]. Numerous reviews have documented the nutritional value, phytochemistry, and pharmacological properties of *Passiflora* species [5,6]. Passion fruit is rich in bioactive compounds, including polysaccharides, carotenoids, polyphenols, and flavonoids, and exhibits diverse health-promoting properties such as antioxidant, anti-inflammatory, antidiabetic, and antitumor activities [7,8,9,10]. Both the fresh fruit and its juice are consumed as dietary sources of essential nutrients and functional components [11,12].

However, as the passion fruit processing industry continues to evolve, a critical issue that warrants attention is the disposal of by-products generated during this process. Globally, approximately 40% of passion fruit is utilised for industrial processing. The peel that is produced during this process accounts for approximately 50% to 60% of the fruit’s total weight, thus representing a significant by-product. Drawing upon the example of China, it is evident that the substantial processing volume results in the generation of nearly one million tonnes of peel on an annual basis. PFP constitutes the primary by-product and retains a substantial proportion of the active components inherent in passion fruit. It is rich in bioactive compounds, including polyphenolic substances (e.g., gallic acid), acidic polysaccharides (e.g., pectin), flavonoids, and natural pigments [13,14,15]. The recovery and utilization of PFP present significant application potential within the food industry—such as in pectin extraction and functional food development—as well as in the creation of bio-based materials [16]. Of particular importance is the prospect for further utilizing PFP as part of a promising sustainable trend aimed at enhancing the value of agro-industrial by-products. Furthermore, research into the development and utilization of food processing by-products has emerged as a prominent area both domestically and internationally. Recent literature has reviewed various aspects related to such by-products—including apple pomace and banana peel—which have found extensive applications across both food and pharmaceutical industries [17,18]. Additionally, recent studies have demonstrated that certain industrial by-products, such as apple pomace and sugarcane bagasse, can be effectively utilized in producing bread, confectionery items, and extruded snacks [19].

Despite the extensive research conducted on the functional characteristics and health effects of PFP, a key research limitation remains the insufficient understanding of its bioavailability. This limitation also constrains its innovative applications in the food sector. Consequently, there is an urgent need for a comprehensive analysis of the health benefits associated with the active components found in PFP as well as their bioavailability. In summary, this paper provides an overview of extraction methods, active characteristics, bioavailability, and practical applications of nutritional components derived from PFP in food. It also discusses the limitations and challenges encountered in the development of PFP while ultimately proposing a promising strategy for future development. As a valuable resource with significant potential, the comprehensive utilization of PFP components is particularly crucial. The proposed utilization method represents a promising approach that merits further attention, research, and practical application in future endeavors.

## 2. Nutrients

PFP is rich in a diverse array of nutrients, particularly vitamins, DF, and minerals. These components can provide the human body with essential nutrients while serving as a cost-effective source of nutrition. The nutritional composition of PFP is presented in Table 1.

### 2.1. Carbohydrates and Dietary Fiber

Carbohydrates are of pivotal significance in the provision of energy to the human body. Fiber polysaccharides represent a category of complex carbohydrates that are present in plant-based foods. These polysaccharides are composed of dietary fibers that are recalcitrant to human enzymes, including cellulose, hemicellulose, pectin, and β-glucan. While these non-starch polysaccharides do not directly provide energy, they are nevertheless essential for digestive health and can also regulate blood sugar and cholesterol levels [20]. Xylitol, a widely used sugar substitute, has also been demonstrated to offer numerous health advantages [21]. Infante-Neta et al. [22] identified that PFP contains substantial amounts of cellulose, which can be utilized for xylitol production. By optimizing the production conditions, the yield of xylitol was increased to 14.97 g/L, thereby establishing it as a highly promising raw material for xylitol synthesis. Furthermore, Guimarães et al. [23] reported that PFP contains reducing sugars at a concentration of 3.06 ± 0.12 g/100 g.

DF is widely acknowledged as the seventh essential nutrient, playing a crucial role in human physiological functions through mechanisms such as the regulation of gut microbiota and the enhancement of satiety [24]. PFP serves as a raw material that is rich in DF, with studies reporting levels reaching up to 71.79 g/100 g [8]. Liu et al. [25] discovered that the SDF content in PFP amounted to 20.51%, demonstrating significant physiological benefits. In addition, Fonseca et al. [26] reported that purple passion fruit peel (PPFP) contains an exceptionally high level of DF, with concentrations reaching 577 g/kg. Presently, research on the extraction of DF from PFP is underway, and a substantial number of clinical trials have validated the efficacy of DF in addressing obesity and diabetes while promoting gut health. However, further comprehensive research is necessary to validate any potential side effects or interactions with other medications.

### 2.2. Vitamins

Vitamins are essential nutrients required by the human body and play a crucial role in vital bodily functions [27]. Notably, the vitamins found in passion fruit are predominantly present in the juice, with lower concentrations detected in the peel. In a study conducted by Dos Reis et al. [28], a comparison was made between the vitamin A (VA) content of yellow passion fruit peel (YPFP) and PPFP. The results indicated that PPFP exhibited the highest concentration of VA, averaging 59.69 ± 2.55 μg/100 g. Furthermore, Guimarães et al. [23] reported that PPFP contains 28.07 ± 0.77 mg of vitamin C (VC) /100 g. These findings suggest that incorporating PPFP into food formulations may aid in achieving the recommended daily intake of 45 mg as established by WHO/FAO.

### 2.3. Minerals, Lipids, and Proteins

In addition to common carbohydrates, DF, and lipids, PFP also contains small amounts of proteins, as well as minerals and trace elements. The protein and lipids present in passion fruit are predominantly located in the seeds, with only negligible quantities found in PFP. Gamarra-Castillo et al. [29] reported a protein content of 7.571 ± 0.232%. In contrast, do Prado Ferreira and Tarley [30] determined that PFP contained 8410 mg/100 g of total lipids. Furthermore, Dos Reis et al. [28] conducted a comprehensive analysis of the mineral composition of YPFP and PPFP, revealing that YPFP exhibited higher concentrations of phosphorus (P) and sulfur (S) compared to PPFP; conversely, PPFP demonstrated elevated levels of sodium (Na), magnesium (Mg), potassium (K), Zinc (Zn), and calcium (Ca) (see Table 1). This discrepancy may be attributed to differences in varietal composition. To date, there has been a paucity of research on the nutritional distinctions between PPFP and YPFP. Therefore, exploring the differences between PFP from various species or color variants is essential for the broader application of PFP.

**Table 1:** Nutrient Content in PFP.

Nutrient	Origin	Content	References
Carbohydrate	YPFP	85.78 ± 0.00 g/100 g	[27]
	PPFP	80.71 ± 0.00 g/100 g	[27]
	PPFP	76 g/kg	[25]
	PPFP	78.267 ± 0.517%	[29]
DF	YPFP	45.18 ± 0.83 g/100 g	[13]
	YPFP	61.16 ± 1.02 g/100 g	[27]
	PPFP	61.68 ± 1.31 g/100 g	[27]
	PPFP	577 g/kg	[25]
	PPFP	62.459 ± 2.857%	[29]
	YPFP	69.69 ± 0.88 g/100 g	[31]
VA	YPFP	22.71 ± 0.98 μg/100 g	[27]
	PPFP	59.69 ± 2.55 μg/100 g	[27]
VC	PPFP	4.58 g/kg	[25]
P	YPFP	140 ± 1.30 mg/100 g	[27]
	PPFP	70,00 ± 1.12 mg/100 g	[27]
	PFP	240 ± 1.71 mg/100 g	[27]
S	YPFP	7000 ± 0.40 mg/100 g	[27]
	PPFP	160 ± 1.35 mg/100 g	[27]
Na	YPFP	2.20 ± 0.02 mg/100 g	[27]
	PPFP	7.30 ± 0.12 mg/100 g	[27]
	PPFP	54.107 mg/kg	[29]
Mg	YPFP	120 ± 0.90 mg/100 g	[27]
	PPFP	130 ± 0.97 mg/100 g	[27]
	PPFP	1.60 g/kg	[25]
	PPFP	836.964 mg/kg	[29]
K	YPFP	2600 ± 15.7 mg/100 g	[27]
	PPFP	2800 ± 16.3 mg/100 g	[27]
	PPFP	31,065.357 mg/kg	[29]
Ca	YPFP	250 ± 1.98 mg/100 g	[27]
	PPFP	310 ± 1.69 mg/100 g	[27]
	PPFP	2833.036 mg/kg	[29]
	YPFP	0.226 g/100 g	[31]
Zn	PPFP	6.071 mg/kg	[30]
Lipid	YPFP	4.20 ± 0.02 g/100 g	[13]
	PFP	3.47 ± 0.3 g/100 g	[29]
	PPFP	6 g/kg	[25]
	PFP	8410 mg/100 g	[29]
Protein	YPFP	3.14 ± 0.31 g/100 g	[13]
	YPFP	3.40 ± 0.06 g/100 g	[23]
	PPFP	6.47 ± 0.04 g/100 g	[27]
	PFP	8.41 ± 0.1 g/100 g	[29]
	PPFP	34 g/kg	[25]
	PPFP	7.571 ± 0.232%	[29]

## 3. Phytochemical Composition

PFP is a rich source of bioactive compounds, rendering it an excellent candidate for various applications. Understanding the functions of these compounds is crucial for optimizing their utilization across different industrial sectors. Therefore, it is essential to develop a comprehensive understanding of the mechanisms that underlie the bioactive properties of PFP. These compounds include polyphenols such as gallic acid and neochlorogenic acid, as well as flavonoids and carotenoids like β-carotene and lutein. Table 2 outlines the composition of PFP’s active components.

### 3.1. Polysaccharides

Polysaccharides encompass pectin, cellulose, hemicellulose, and other compounds. Pectin is a heteropolysaccharide with diverse industrial applications in sectors such as food, agriculture, medicine, and biomedicine [32]. Galacturonic acid constitutes the primary component of pectin from PFP, representing approximately 65% of the total pectin content. It can be classified into high-methoxy pectin (HMP) and low-methoxy pectin (LMP) based on the degree of methylation. HMP undergoes conversion to LMP through deesterification—a process that is crucial for minimizing sugar usage in food applications while also influencing the physiological functions of pectin [33,34,35]. Teng et al. [36] conducted an analysis of the composition of pectic polysaccharides extracted from PPFP using two distinct methods via gas chromatography. The study revealed that the monosaccharide composition predominantly includes galacturonic acid, glucose, xylose, arabinose, galactose, and rhamnose. Moreover, the findings indicated that different extraction methods significantly affect monosaccharide composition; specifically, pectin extracted using ultrasonic-assisted techniques exhibited elevated levels of galacturonic acid along with enhanced antioxidant and anti-inflammatory activities. This phenomenon is attributed to the ultrasonic-assisted extraction method’s ability to better preserve the activity of heat-sensitive compounds. Teles et al. [37] found that the majority of pectin components extracted from YPFP were primarily composed of high galacturonic acid polysaccharides (HG), rhamnogalacturonic acid polysaccharide I (RGI), rhamnogalacturonic acid polysaccharide II (RGII), and xylogalacturonic acid polysaccharides (XG). A comparative analysis of the pectin composition in YPFP and PPFP with that reported by Teng et al. [36] indicates a significant degree of similarity. Furthermore, Teles et al. [37] quantified cellulose content at 51.99% and hemicellulose at 18.93% in PFP. Liang et al. [38] utilized ultrasound-assisted extraction techniques to extract PPFP, resulting in pectin with increased total pectin content and reduced methylation levels, thereby enhancing the stability of the extracted pectin. Concurrently, Zhao et al. [39] and Liang et al. [38] demonstrated that variations in extraction methodologies can lead to changes in both structural and physical properties of pectin within PFP, significantly influencing its practical applications. Consequently, developing appropriate extraction methodologies is essential for advancing the utilization of pectin.

### 3.2. Total Phenolics (TPC) and Total Flavonoid (TFC) Compounds

Vo et al. [40] demonstrated that the combination of natural deep eutectic solvents (NADES) with ultrasound-assisted extraction (UAE) or microwave-assisted extraction (MAE) technologies can effectively extract phenolic and terpenoid compounds from PFP. In comparison to their previous methods employing UAE or MAE in isolation [41], the use of NADES resulted in a significant enhancement in extraction efficiency. Under optimized UAE conditions, the yields of total terpenoids (TTC) and TPC reached 56.9 mg ursolic acid (UA)/g dry weight (dw) and 21.03 mg gallic acid equivalent (GAE)/g dw, respectively. Under optimized MAE conditions, TTC and TPC were recorded at 32.82 mg UA/g dw and 22.12 mg GAE/g dw, respectively. Huo et al. [42] further employed NADES to optimize both UAE and MAE processes, successfully isolating various phenolic compounds from YPFP, including gallocatechin, epicatechin, and myricetin. Among these compounds, gallic acid was identified as the most abundant, with a concentration of 26.29 μg/g. Notably, the TPC of PPFP was found to be significantly higher than that of other parts of the fruit, highlighting its potential as a valuable source for phenolic enrichment. Furthermore, Siniawska and Wojdyło [43] isolated 51 polyphenolic compounds from PPFP using LC-QTOF/ESI-MS. The identified compounds primarily included flavonoids (25 compounds, accounting for 52%), flavanols (8 compounds, accounting for 16%), flavan-3-ols (6 compounds, accounting for 7%), phenolic acids (4 compounds, accounting for 3%), and anthocyanins (7 types, accounting for 21%). Da Costa et al. [44] further observed that PPFP exhibits the highest polyphenol content during its mature stage, with chlorogenic acid levels reaching 45.04 ± 6.49 mg/100 g and catechin at 11.39 mg/100 g. These concentrations surpass those found in seeds and pulp, thereby confirming that PPFP serves as a significant reservoir of phenolic compounds. This conclusion was further supported by Fonseca et al. [26], who detected TPC levels ranging from 225.6 to 255.3 mg GAE/100 g in PPFP, a level significantly higher than that observed in other parts, accompanied by high antioxidant activity. Additionally, a substantial body of research has corroborated the diversity and functionality of phenolic compounds present in passion fruit. Dominguez-Rodriguez et al. [45] employed a pressurized hot water extraction method to identify a total of 57 phenolic compounds across four varieties of passion fruit, with TPC values ranging from 5.08 to 30.19 mg GAE/g. Carmona-Hernandez et al. [46] identified 16 major compounds in their study, including phenolic acids, flavonoids, catechins, and anthocyanins (notably catechin, epicatechin, and ferulic acid). They confirmed that these extracts can mitigate intestinal damage by inhibiting inflammation. Furthermore, Guimarães et al. [23] demonstrated that the phenolic content in passion fruit peel reaches 19.94 mg GAE/100 g and exhibits an impressive antioxidant capacity of 87.07%, thereby underscoring its significant physiological functional value. In summary, passion fruit peel can be regarded as an excellent source of phenolic compounds with beneficial physiological functions for human health.

### 3.3. Natural Pigments

The natural pigment content in PFP is significantly higher than in the fruit pulp, with cyanidin-3-glucoside (C3G) being the primary anthocyanin component. Research evidence indicates that Gamarra-Castillo et al. [29] found that the anthocyanin content of dried passion fruit peel (0.156 mg C3G/g) was significantly lower than that of fresh peel (0.535 mg C3G/g), suggesting that thermal drying significantly degrades anthocyanins. This aligns with the instability of anthocyanins at high temperatures—high temperatures can disrupt their molecular structure, leading to the loss of antioxidant activity. Ghada et al. [47] optimized the extraction process using ethanol as a solvent, achieving an anthocyanin yield of 9 ± 1 mg C3G/g from PPFP. Additionally, Kawasoe et al. [48] further identified the core pigments in PPFP as including: C3G (9.8 μg/10 mg freeze-dried sample), delphinidin-3-glucoside, and cyanidin derivatives (such as cyanidin-3-rhamnoside). The aforementioned study confirmed that pigments extracted from passion fruit peel exhibit excellent stability under light and heat treatment, making them suitable for use in jelly coloring. The coloring effect achieved through this method is comparable to that of synthetic pigments, while also contributing to the reduction of food waste (such as peels) and lowering production costs. Dos Reis et al. [28] conducted a comparative analysis of three distinct types of passion fruit, revealing that those with an orange hue exhibited the highest total carotenoid content, with β-carotene levels reaching 21,274 ± 676 μg/100 g—significantly higher than those observed in yellow and purple varieties (*p* < 0.05). Furthermore, components such as lutein and zeaxanthin demonstrated accumulation patterns specific to each variety. In summary, passion fruit peel has been identified as a sustainable source of C3G—a natural pigment with considerable commercial value due to its high color intensity combined with notable biological activity, including antioxidant properties. This characteristic positions it as a promising natural alternative to synthetic dyes while highlighting the significant potential for high-value utilization of agricultural byproducts.

**Table 2:** Content of bioactive components in PFP.

Bioactive Ingredients	Origin	Extraction	Content	References
Pectin	YPFP	Acid extraction	37.67 ± 0.97 g/100 g	[28]
	PPFP	Acid extraction	32.85 ± 1.20 g/100 g	[28]
	PPFP	Ultrasound-assisted conventional extraction	12.67%	[28]
	PFP	High-pressure heating and conventional heating	14.34%	[49]
	PFP	Enzymatic extraction	26 g/100 g	[50]
	PFP	Subcritical water and pressurized natural deep eutectic solvents	15.70%	[51]
	PFP	Magnetic induction electric field treatment to assist three-phase distribution	6.58%	[52]
TPC	PPFP	Organic solvent extraction	24 ± 1 mg GAE/g	[26]
	PFP	Ultrasound-assisted pressurized liquid extraction	2.07 ±0.05 mg GAE/g	[53]
Gallic acid	PPFP	L-Proline: Citric Acid (Pro-CA) Extract	8.22 ± 0.15 μg/g	[42]
Epicatechin	PPFP	L-Proline: Citric Acid (Pro-CA) Extract	2.74 ± 0.08 μg/g	[42]
Quercetin	YPFP	Acid extraction	760.21 ± 32.07 mg/100 g	[28]
	PPFP	L-Proline: Citric Acid (Pro-CA) Extract	1.57 ±0.14 μg/g	[42]
Rutin	PPFP	L-Proline: Citric Acid (Pro-CA) Extract	6.66 ± 0.73 μg/g	[42]
Carotenoid	PFP	Ultrasound-assisted extraction of vegetable oils	1176.195 μg/100 g	[54]
	YPFP	Organic solvent extraction	918.41 ± 36.81 μg/100 g	[28]
	PPFP	Organic solvent extraction	1244 ± 52.5 μg/100 g	[28]
Anthocyanin	PPFP	Acid extraction	103,686.48 ± 542.11 μg/100 g	[28]
	PPFP	Microwave-assisted extraction	0.156 ± 0.0024 mg C3G/g	[29]
	PPFP	Solvent extraction	577.59 mg C3G 100/g	[55]
Cellulose nanocrystals	PFP	Acid extraction	58.1 ± 1.7%	[56]

## 4. Biological Effects

Plants have been shown to be rich in various bioactive substances, including phenolic compounds, carotenoids, and polysaccharides. These substances have been demonstrated to possess a variety of significant physiological regulatory functions in the human body, including substantial antioxidant activity, anti-inflammatory effects, and the capacity to enhance intestinal health. The biological effects of PFP are shown in Table 3.

### 4.1. Antioxidant

**Figure 1:**
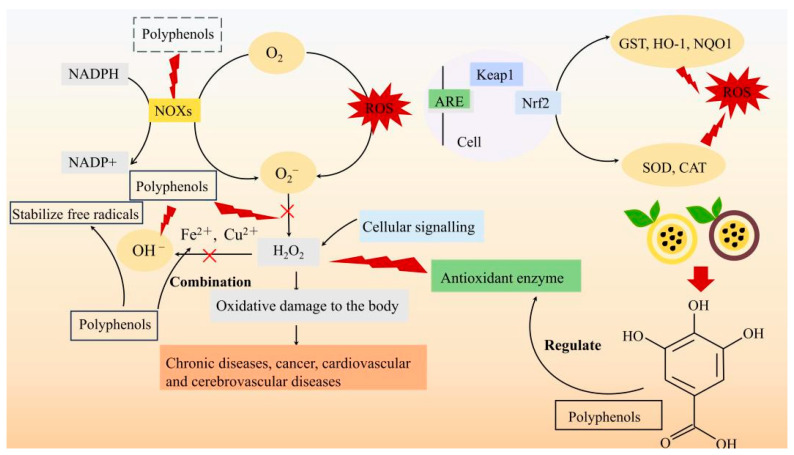
Antioxidant mechanisms of polyphenols in PFP.

During the processes of growth and development, ROS are frequently generated. These ROS have the potential to damage cells, thereby accelerating cellular aging and contributing to oxidative stress. Excessive levels of ROS have been shown to have detrimental effects on human health, including the onset of cancer and cardiovascular diseases [57,58]. Consequently, the scavenging of ROS is a critical factor in maintaining human health. PFP contains a diverse array of phenolic compounds and flavonoids that exhibit significant antioxidant capacity. Da Costa et al. [44] identified that PFP comprises catechins, chlorogenic acid, gallic acid, resveratrol, caffeic acid, ferulic acid, among other polyphenols and flavonoids capable of effectively scavenging reactive oxygen species [59]. In addition to phenolic compounds and flavonoids, certain pectin polysaccharides have demonstrated remarkable antioxidant properties. Teng et al. [36] reported that two novel pectin polysaccharides, PFSP60 and UPFSP60, extracted from PPFP significantly enhanced the scavenging capacity for DPPH, ABTS, and superoxide anion radicals. Notably, the antioxidant activity increased with rising concentrations of these polysaccharides. Furthermore, the monosaccharide composition and glycosidic bonds of these two distinct polysaccharides were found to differ. In summary, UPFSP60 exhibited a higher galacturonic acid content and lower molecular weight compared to PFSP60, resulting in stronger antioxidant and anti-inflammatory activities. Outama et al. [60] conducted a study investigating the effects of PFP on the immune system and antioxidant activity in Nile tilapia. Following administration of PSPP20 at a dosage of 20 g/kg over periods of 4 and 8 weeks, significant differences were observed in the expression levels of immune- and antioxidant-related genes such as lbp, gst-α, and gpx. Notably, PSPP20 displayed the highest expression levels among all tested samples. Dos Reis et al. [28] evaluated the antioxidant capacity of PFP of various colors using methods such as ABTS and DPPH, revealing that YPFP exhibited a significantly higher antioxidant capacity compared to orange PFP and PPFP. Dominguez-Rodriguez et al. [45] identified 57 phenolic compounds, among which flavonoids, chalcones, and phenolic acids may be critical determinants of the antioxidant capacity of PFP, demonstrating substantial antioxidant activity. Figure 1 illustrates the antioxidant mechanisms of polyphenols in PFP. In summary, PFP possesses remarkable antioxidant capacity and high free radical scavenging rates, rendering it an appropriate raw material for pharmaceuticals or functional foods.

### 4.2. Anti-Inflammatory

**Figure 2:**
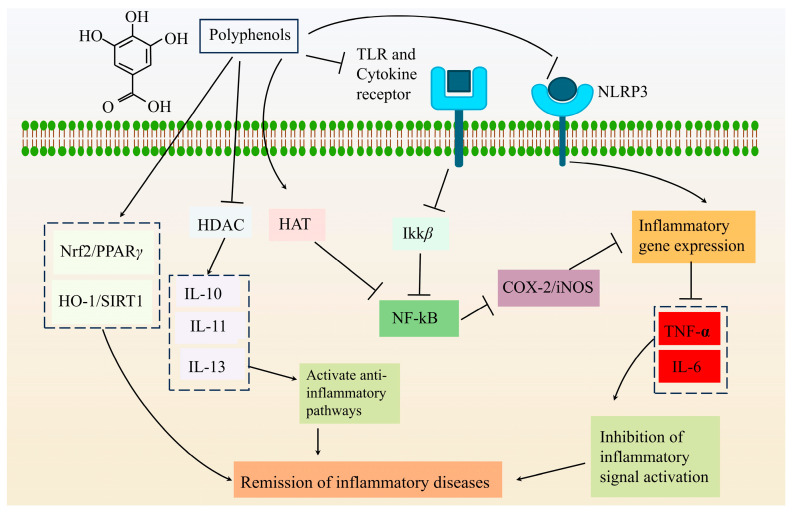
Mechanism of polyphenols’ effect on inflammation.

Recent studies have shed light on the anti-inflammatory properties of PFP. Belmonte-Herrera et al. [61] demonstrated that PFP treatment (8 mg/mL in drinking water) effectively reduced colitis-induced damage in female C57BL/6J mice. Their biochemical and molecular analyses revealed that PFP not only suppressed pro-inflammatory cytokine expression but also strengthened the intestinal protective barrier. Interestingly, the treatment also boosted short-chain fatty acid production—these metabolites are known to play a crucial role in maintaining colonic homeostasis. Since microbial imbalance can trigger immune responses leading to mucosal damage and intestinal inflammation, these findings strongly support PFP’s prebiotic potential (Figure 2). Building on this, Teng et al. [36] identified two novel pectic polysaccharides (PFSP60 and UPFSP60) in PFP that exhibited both antioxidant and anti-inflammatory activities by downregulating pro-inflammatory factors. Meanwhile, Siniawska & Wojdyło [43] took the research further by isolating 51 bioactive compounds from PFP extracts, confirming through in vitro studies their significant anti-inflammatory and antioxidant effects. While the collective evidence clearly positions PFP as a potent anti-inflammatory agent due to its rich bioactive profile, we still don’t fully understand how it modulates human inflammatory factors. This knowledge gap presents an exciting avenue for future research.

### 4.3. Improvement of Intestinal Health

**Figure 3:**
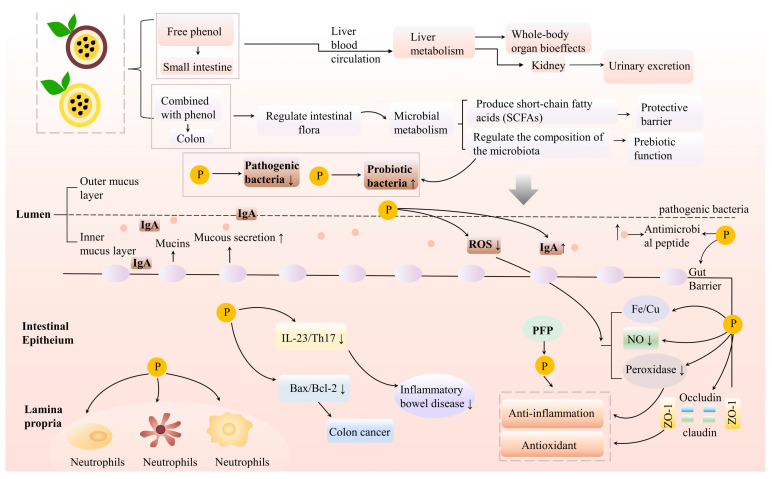
Protective effect of polyphenols in PFP on intestinal health. P stands for polyphenols in the figure.

In the study conducted by Yu, Wu et al. [14], pectic polysaccharides (PFP-T and PFP-UM) were extracted from YPFP using three-phase partitioning and ultrasonic–microwave synergistic three-phase partitioning, respectively. The in vitro simulated digestion experiment revealed an increase in the total relative abundance of the intestinal flora in the phylum of thick-walled bacteria and Bacteroidetes. Compared to PFP-UM, PFP-T exhibited a superior capacity to promote the proliferation of beneficial bacteria, including *Prevotella*, *Eubacterium*, and *Ruminococcus*. Simultaneously, it inhibited the growth of potentially harmful bacteria such as *Escherichia coli* and *Shigella* while enhancing short-chain fatty acid production. In addition, Lubis et al. [62] investigated the effects of adding pectin to feed on the intestinal health of Nile tilapia (*Oreochromis niloticus*) and found that the addition of pectin not only had positive effects on the growth performance, enhancement of innate immunity, up-regulation of antioxidants and expression of immune-related genes, but also included positive changes in intestinal morphology and intestinal microbiota (Figure 3).

Jiang et al. [63] also found the same finding that passion fruit improves gut health from multiple perspectives. Ju et al. [64] showed better results relative to PFP by adding 3% enzymatically PFP (EFP) contrast to PFP in the feed of triple-yellow chickens, which, by ameliorating inflammation, could improve the health of broiler chickens by increasing the diversity of microorganisms in the cecum. Furthermore, both PFP and EPF supplementation promoted serum antioxidant status and anti-inflammatory activity to varying degrees. This finding was confirmed after analysis of the gut microbiota and metabolites, where antioxidant-related metabolites (ganoderic acid, γ-CEHC, S-adenosylmethionine), anti-inflammatory-related metabolites (OEA), and anti-inflammatory-associated bacteria (Butyricaceae) were found to be increased with EPF supplementation. Moreover, the addition of 3% EPF to the diet had a positive effect on the biosynthesis of phenylpropanes, which is strongly associated with antioxidant and anti-inflammatory properties. This was also confirmed by Pimisa, Prasongsuk et al. [65] through in vitro experiments on the potential of PFPEP and the combination of PFP/probiotics in modulating the intestinal microbiota and its metabolic activity in healthy adults, when added to PFP and PFP/probiotics.

In their analysis of the reparative effects of SDF extracted from PFP, da Silva et al. [66] found that SDF could combine with 5-FU to have a therapeutic effect on intestinal mucositis. They conducted mouse experiments to ascertain whether SDF preserved 5-FU-induced colonic length and histological damage. The results of these experiments demonstrated that SDF significantly restored 5-FU-induced intestinal oxidative stress and inflammation, as well as the enlargement and swelling of the spleen. In contrast, da Silveira et al. [67] conducted experimental research on mice, in which the animals were orally fed pectin fibres extracted from YPFP. The results of this study indicated that this led to an effect on the intestinal barrier and a worsening of sepsis in mice. In this study, despite the capacity of YPF to modulate inflammation (by increasing PELF IL-6 and small intestinal IL-10 and decreasing small intestinal TNF-α production and leukocyte accumulation in the peritoneum) and oxidative stress (by attenuating PELF LPO and TAC levels), the fibres were unable to prevent sepsis-induced mortality and hypothermia. Instead, they accelerated mortality and hypothermia in mice with infectious disease. This paradoxical result may be due to a slower disruption of the intestinal barrier [68].

Xu et al. [69] selected PFP fermentation extract, which increased gastrointestinal transport capacity and fecal water content, protected the structural integrity of colonic tissues, and reduced inflammatory infiltration. They demonstrated that its active ingredient, Kae, had preventive and relieving effects on constipation and hemorrhoids. The precise mechanisms by which PFP and its active ingredient Kae regulate the ESR1 and PI3K/Akt pathways to ameliorate constipation and hemorrhoids remain to be elucidated. Further exploration into the mechanisms of other active ingredients is warranted to ascertain their potential to improve constipation.

### 4.4. Additional Biological Effects

Fonseca et al. [5] identified gamma-aminobutyric acid (GABA) in PPF methanol extracts, which is associated with antihypertensive activity, with concentrations ranging from 2.40 to 4.40 mg/g. Diabetes is a chronic endocrine disorder that disrupts normal metabolic processes and bodily functions. For many years, it has been hypothesized that plant-based products rich in bioactive compounds (such as phenolic compounds, coumarins, and terpenoids) have the potential to lower blood glucose levels [70,71]. Siniawska & Wojdyło [43] demonstrated that a mixture of PPFP compounds exhibited inhibitory effects on enzyme activity by inhibiting α-amylase, α-glucosidase, and lipase; this plays a significant role in the prevention and treatment of obesity. Similarly, Vuolo et al. [72] reported that PFP can decrease inflammatory cytokines in serum, mitigate oxidative stress, and alleviate chronic inflammatory responses as well as fat deposition. Cabral et al. [73] employed liquid chromatography-triple quadrupole mass spectrometry (LC-QqQ-MS/MS) to identify five C-glycosylated flavonoids—visenin-2, kaempferol, isokaempferol, quercetin, and isoquercetin—in extracts derived from PFP. When compared to insulin monotherapy, the combination of the extract with insulin as an adjunctive therapy exhibited a significant improvement in glycemic control within a 60-day period (*p* < 0.05). Furthermore, this combination was found to prevent renal and cardiac complications in rats with type 1 diabetes. De Oliveira Balthar et al. [74] also reported that pectin and fiber extracted from PFP can be effective in treating type 2 diabetes. PFP contains soluble fibers such as pectin, which enhance DF intake and contribute to reductions in glucose levels and circulating lipids among diabetic patients. Hu et al. [75] investigated the anti-fatigue properties of anthocyanins purified from PPFP in mice. Their results indicated that PFE significantly extended the duration of forced swimming under load while simultaneously reducing levels of lactate dehydrogenase (LDH), blood lactate (BLA), and blood urea nitrogen (BUN). Additionally, it was observed that liver glycogen (LG) content increased following treatment with PPFP. Moreover, PPFP has been shown to mitigate fluctuations in oxidative stress biomarkers such as malondialdehyde (MDA), antioxidant enzymes including superoxide dismutase (SOD), and inflammatory cytokines like TNF-α, IL-1β, and IL-6. Furthermore, PFEA has been shown to upregulate the mRNA expression of PGC-1α and PPARα in skeletal muscle. In summary, the findings of this study indicate that supplementation with PFEA can mitigate fatigue effects. Nerdy and Ritarwan [76] reported that the hepatoprotective and nephroprotective activities of PPFP extract were superior to those observed with red passion fruit peel extract and YPFP extract. The study also demonstrated that the hepatoprotective effects of PFP are linked to its phytochemical constituents, including flavonoids and polyphenols. Additionally, PFP has been found to exhibit a range of physiological activities, such as anti-aging properties, memory enhancement, antibacterial effects, and acceleration of wound healing [77,78,79,80].

**Table 3:** Biological Effects in PFP.

Physiological Function	Research Material	Research Type	Mechanism of Action	References
Antioxidant	PFP	In vitro study	Eliminate ROS, such as DPPH, ABTS, and superoxide anion radicals	[44]
	PFSP60, UPFSP60	In vitro study	Enhance the ability to scavenge free radicals	[36]
	PFP	Animal experiment	Up-regulate the expression of antioxidant genes	[60]
Anti-inflammatory	PFPF	Animal experiment	Inhibit pro-inflammatory cytokines, enhance the intestinal barrier, and increase short-chain fatty acids	[61]
	PFSP60, UPFSP60	In vitro study	Down-regulate the expression of pro-inflammatory factors	[36]
	PFP	In vitro study	It has anti-inflammatory and antioxidant activities	[43]
Improve intestinal health	YPFP	In vitro digestion simulation	Promote beneficial bacteria (*Prevosiella*, *Megalococcus*) and inhibit harmful bacteria (*Escherichia*, *Shigella*)	[14]
	YPFP	Animal experiment	Improve intestinal morphology and microbiota, and enhance immunity	[62]
	PFPF	Animal experiment	Increase the diversity of cecal microorganisms and enhance antioxidant and anti-inflammatory metabolic properties	[64]
	PFP	Animal experiment	Relieve intestinal mucositis caused by 5-FU, reduce oxidative stress and inflammation	[66]
	PFP	Animal experiment	Improve constipation, protect colon structure, and reduce inflammatory infiltration	[69]
Lower blood sugar	PFP	In vitro study	Inhibit α-amylase, α-glucosidase and pancreatic lipase	[43]
	PFP	Animal experiment	Improve blood sugar control and protect heart and kidney functions	[73]
	PFP	Animal experiment	Lower blood sugar and circulating lipids	[74]
Anti-fatigue	PPFP	Animal experiment	Reduce lactic acid and urea nitrogen, increase liver glycogen, and improve oxidative stress and inflammation	[75]
Liver protection and kidney protection	PPFP	Animal experiment	It is superior to other colors of PFP and is related to the content of polyphenols and flavonoids	[76]
Other functions	PFP	A variety of studies	Anti-aging, improve memory, antibacterial and so on	[77,78,79]

## 5. Bioavailability/Bioaccessibility

In the field of pharmacology, bioavailability represents a multifaceted concept that encompasses both the rate of gastrointestinal transport and the extent to which a substance is absorbed into systemic circulation. The U.S. Food and Drug Administration (FDA) provides a detailed definition of bioavailability: “Bioavailability refers to the rate and extent to which the active ingredient or active portion of a drug is absorbed and reaches the site of action” [81]. It is essential to recognize that assessments of bioavailability can be performed in vitro using specialized cell lines or in vivo utilizing animal models and human biological fluids [82].

Cao et al. [83] conducted an in vitro gastrointestinal simulation digestion study to assess the residual content of phenolic compounds in PPFP extracts. The results indicated a significant reduction in TPC, TFC, and anthocyanin content following simulated digestion. Notably, the levels of TPC and flavonoids that were retained and reached the colon after digestion were substantially higher than those absorbed in the small intestine; specifically, the BI value (the amount absorbed in the small intestine) was less than 20%, while the RID value (the amount reaching the colon post-digestion) exceeded four times that of the BI value. Although the bioavailability of phenolic compounds entering the dialysis bag is low, their antioxidant capacity may offer protective effects on damaged pancreatic cells. PFP extracts have demonstrated considerable antioxidant capacity as well as α-glucosidase inhibitory activity. Furthermore, research has shown that fermentation enhances the bioavailability of peel components. Nguyen et al. [84] measured the phenolic content of PFP extracts and found that after fermentation with Aspergillus niger, their TPC was four times greater than that of unfermented extracts. Subsequent in vitro simulated digestion tests revealed increased phenolic content and bioavailability for fermented peel compared to its unfermented counterpart, suggesting that fermented PFP holds promise as a novel functional food. De Souza et al. [85] investigated the in vitro digestibility of PFP and found that, compared to other samples, PFP exhibited a capacity to delay glucose absorption in the body. Do Prado Ferreira and Tarley [33] analyzed the bioavailability of PFP during saliva, gastric, and gastrointestinal phases through in vitro simulated digestion experiments. They also examined the bioavailability of minerals (Mn, Zn, Cu, Fe, Ca, and Mg) present in flour post-digestion. The bioavailability of various elements differs significantly; this variation may be attributed to chemical bonds and specific chemical reactions involved. Following gastrointestinal digestion, Mg, Mn, and Fe were identified as contributing most substantially to bioavailable concentrations at 14%, 52%, and 12%, respectively.

## 6. The Application in the Food Industry

The various bioactive properties of PFP establish its potential as a functional food ingredient. The addition of precise quantities of PFP to foodstuffs has been demonstrated to effect alterations in their characteristics. Consequently, this section offers a synopsis of the potential of PFP in food-related applications. The utilization of PFP in the domain of food is delineated in Table 4.

### 6.1. Wheat Flour Foods

Passion fruit peel powder, which is rich in dietary fibre and polyphenolic compounds, demonstrates significant potential as a high-performance nutritional fortifier and natural functional ingredient in flour-based foods. A plethora of studies have not only corroborated its feasibility for addition, but have also delved deeper into its mechanisms of action and unique advantages.

In the domain of baked goods, Macedo et al. [86] proposed a methodologically innovative approach. Yellow passion fruit peel powder was incorporated into flour, and cookies and cakes made from this mixture exhibited high DPPH radical scavenging activity, with a total phenolic content of 645.54 mg GAE/100 g. A significant advancement was the employment of paper spray mass spectrometry, a method that facilitated the systematic identification of 22 organic compounds in the fortified flour. This study provides a comprehensive chemical analysis of the bioactive compounds present in passion fruit peel, thereby offering substantial scientific evidence for enhancing the nutritional value of baked goods. In a similar vein, Sampaio et al. [87] incorporated varying amounts of PFP (8.5–17%) into cookies to assess its impact on sensory quality, enhancing the nutritional value of the product and yielding higher sensory quality compared to conventional cookies. The albedo-rich regions, which correspond to white portions of the fruit, respectively, have been identified as promising low-cost DF additives due to their versatility in various applications. Beyond the realm of nutritional enhancement, the functional properties of PFP merit equal consideration. Ning et al. [88] conducted a study to ascertain the effects of purple passion fruit peel powder on the characteristics of biscuit digestion. The findings of the study indicated that the augmentation of PPFP addition exhibited a substantial inhibitory effect on starch hydrolysis in biscuits. This observation suggests the possibility of delaying the elevation of postprandial blood glucose levels. Concurrently, the content of biscuit polyphenols and antioxidant capacity exhibited a linear increase with the addition of PFP, thus elevating its application beyond the scope of dietary fibre supplementation to that of functional food development.

Moreover, PFP incorporation has been demonstrated to enhance the technical properties of products. As demonstrated in the studies conducted by Garcia et al. [89] and Nascimento et al. [90], a significant increase in dietary fibre content was observed in dough and finished products when conventional flour was partially replaced with PFP (up to 30%). Concurrently, these studies indicated that there was an effective reduction in both lipid content and total calories. This development not only fulfils market demands for healthy, low-calorie foods, but also offers novel approaches to addressing dietary needs for individuals with gluten sensitivity or those requiring high-fibre diets. Nasution et al. [91] utilised passion fruit peel extract as a natural antioxidant and antimicrobial agent, combining it with tilapia bone collagen to prepare pancakes. This development led to the successful expansion of PFP’s application boundaries into the domain of food preservation and safety. Its inhibitory effects on Escherichia coli and Staphylococcus aureus demonstrate its potential as a synthetic preservative alternative.

### 6.2. Dairy Products

In the dairy industry, passion fruit peel, notable for its substantial pectin content, functions as a multifunctional additive and fat substitute. It has been demonstrated to enhance texture and flavour in low-fat products, thereby addressing deficiencies in these areas.

As demonstrated by Liu et al. [92], PFP has been shown to enhance both the sensory and nutritional qualities of yoghurt. It was established that the incorporation of passion fruit peel powder into goat milk yogurt resulted in a substantial augmentation of the total phenolic content, total flavonoids, and anthocyanins present within the product. This increase directly led to a notable enhancement in the antioxidant activity of the yogurt. Moreover, optimal addition levels (1%) enhanced the yogurt’s taste and flavour profile, addressing the common issue of negative sensory impacts from functional ingredient additions and providing a reference for developing highly acceptable functional dairy products.

However, more in-depth mechanistic research is provided by the work of Yu et al. [93]. A novel study was conducted in which the effects of passion fruit peel pectin and commercial citrus pectin in low-fat yoghurt were systematically compared for the first time. The findings demonstrated that, even at low addition levels (0.025%), passion fruit peel pectin achieved or surpassed the effects of 0.05% citrus pectin, significantly increasing lactic acid bacteria counts, titratable acidity, and improving yogurt rheology and textural properties. The primary significance of this study is the revelation of its mechanism of action, which is as follows: the pectin from passion fruit peel forms stronger complexes with casein through electrostatic interactions, thereby constructing a more stable protein gel network. This theoretical framework not only elucidates the superior synergistic performance of the solution but also substantiates its technical feasibility as an efficient and economical fat replacement solution. The solution has been shown to maintain excellent product stability throughout a 21-day storage period. Yang et al. [35] further expanded the application prospects of the subject through the modification of pectin. The production of low-methoxyl pectin was achieved through the utilisation of plasma-assisted enzymatic methods, incorporating dielectric barrier discharge plasma. The resulting product exhibited enhanced apparent viscosity, thermal stability, and gelling properties. This modification specifically optimised pectin’s functional characteristics, rendering it particularly suitable for gel-based low-fat dairy products and indicating the potential for developing a new generation of customized food ingredients.

### 6.3. Other Products

Moreover, due to its elevated pectin content, PFP is frequently utilized as a thickening and gelling agent, making it particularly suitable for jelly production [94]. Caroline et al. [95] employed pectin extracted from PFP in the formulation of goji berry jelly, incorporating goji berries as a primary ingredient. Following an extensive sensory evaluation, the overall ratings consistently surpassed 80%, indicating that the product is considered appropriate for both production and consumption. Garrido et al. [96] conducted a series of physicochemical and microbiological analyses on the final product, determining that a combination of 1% PFP and 4% insulin yielded optimal results while remaining within national regulatory limits. Furthermore, Pimisa, Bankeeree et al. [97] discovered that passion fruit sourced from Thailand exhibits a higher degree of esterification, which enhances its suitability for probiotic growth and positively influences sensory quality. Additionally, PFP was found to improve TPC, antioxidant capacity (AOC), and the survival rate of probiotics throughout the storage process in synbiotic ice cream (SIC) products. These findings highlight the potential of PFP as a promising functional ingredient for SIC applications with prospective uses in the food industry or related sectors. Gamarra-Castillo et al. [29] also incorporated PPFP into beverage production to enhance their nutritional value. Their research supports the development of new products characterized by high antioxidant capacity and associated health benefits through the use of PPFP.

### 6.4. Food Packaging

Converting the by-products of passion fruit peels into edible or biodegradable packaging materials is a cutting-edge direction for achieving their high-value utilization and reducing plastic pollution.

The early research by Munhoz et al. [98] affirmed the environmental advantages of using yellow passion fruit by-products to produce films, but also pointed out the challenge of short shelf life due to their high biodegradability. The significance of this work lies in clarifying the core issue that must be addressed in developing such materials: the balance between durability and functionality. Nguyen et al. [99]’s research represents a significant advancement in this direction. They developed cross-linked passion fruit peel pectin/chitosan/pepper leaf composite films for the biodegradable preservation of eggplants. These films not only exhibit satisfactory mechanical properties but, more importantly, demonstrate significant antibacterial activity. The breakthrough of this technology lies in the ingenious integration of the film-forming property of pectin, the antibacterial property of chitosan, and the functionality of plant extracts through multi-component composites and cross-linking techniques, successfully preparing an intelligent packaging material that combines physical protection and biological antibacterial activity, providing a green solution for post-harvest preservation of fruits and vegetables. Currently, the application of PFP in the preservation of fruits and vegetables remains limited; nevertheless, it holds substantial market potential.

### 6.5. Application of High-Value-Added Products

PFP has a dual application: it is utilized in both food production and industrial diesel production. Tarigan et al. [100] demonstrated that PFP can serve as a catalyst for producing diesel at room temperature, thereby reducing the cost of diesel production. Furthermore, PFP has been successfully employed as a heterogeneous catalyst in the synthesis of palm oil biodiesel. Barros et al. [101] reported that calcined PFP possesses a high concentration of potassium (>69%), primarily in the forms of KCl and K_2_CO_3_. A comparative analysis between traditional catalysts and PFP highlights the latter’s significant potential to lower biodiesel production costs, attributed to its derivation from waste materials and its recyclability. Silva et al. [102] assessed the cellulase production by Aspergillus oryzae URM5620 and explored its application as an enzymatic pretreatment for PFP to enhance anaerobic biogas production. The PFase activity of the enzyme produced with 3% substrate and 1% glucose was measured at 1.2 U/mL and 1.7 U/mL CMCase, respectively. The biogas production potential of two hydrolyzates was compared; hydrolyzate 1 yielded 97.02 mL, which is approximately 60% higher than hydrolyzate 2’s yield of 59.9 mL. This finding suggests that optimizing biogas production represents a more achievable goal. These results indicate that fermentation pretreatment utilizing Aspergillus oryzae holds promise for enhancing and accelerating the biological digestion process of lignocellulosic organic waste.

**Table 4:** Applications of PFP in food.

Foods	Addition Amount	Key Findings	References
Flour	100%	DPPH↑, TPC↑, color↓	[86]
Biscuits	8.5–17%	protein↑, ash content↑, DF↑, sensory quality↑	[87]
	30%	DF↑, microbial content↓	[88]
	0–9%	DPPH↑, TPC↑, DF↑, color↓	[89]
	10–30%	vitamins↑, minerals↑, DF↑	[90]
Wrap	1–3 mL	Antioxidant content↑, antibacterial properties↑	[91]
Noodles	0–9%	DPPH↑, TPC↑, DF↑	[11]
Yogurt	0–2.5%	TPC↑, TFC↑, flavonoid glycoside↑, flavor↑	[92]
	0.025%, 0.05%	lactic acid bacteria↑, WHC↑, texture↑, apparent viscosity↑, dynamic viscoelasticity↑, flavor↑	[14]
	0–0.4%	stability↑, lipid↓	[35]
Jelly	1, 3, 5%	Escherichia coli↓, yeast↓, mold↓	[96]
Ice cream	0.4, 0.8%	TPC↑, Antioxidant capacity↑, Probiotic survival rate↑	[97]
Beverage	0.025%	anthocyanin↑	[29]
	73.2 g	DF↑, Antioxidant capacity↑	[103]

Note: In the table, the arrow “↑” indicates an increase (upward adjustment), while “↓” indicates a decrease (downward adjustment).

## 7. Challenges and Future Perspectives

**Figure 4:**
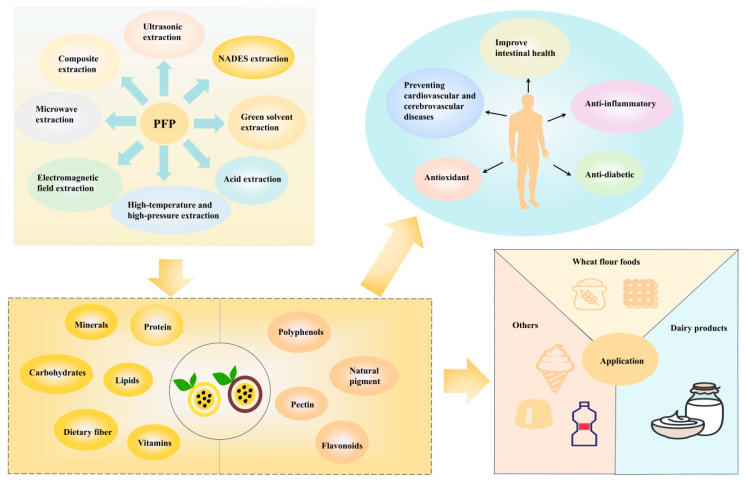
Methods for extracting nutrients from PFP, nutritional components, active ingredients, and their applications.

Despite the promising potential of PFP, several significant challenges hinder its full-scale application and necessitate further research. A summary of the main challenges and the required research focus is presented in Figure 4. Firstly, the instability and low bioavailability of its bioactive compounds present a major obstacle to their effective use. There is an urgent need for more research to elucidate the bioavailability, cellular uptake mechanisms, and precise modes of action of these active ingredients within the host organism. In particular, additional evidence is necessary to clarify the mechanisms by which phenolic compounds, such as flavonoids and chlorogenic acid, exert their health-promoting effects.

Furthermore, current research on the biological effects of PFP remains constrained by numerous limitations. Investigations have predominantly focused on phenotypic changes and gene expression, leaving a significant gap in our understanding of the molecular mechanisms through which these compounds modulate intracellular signaling pathways. Moreover, research on the separation and extraction methods for these substances from PFP remains limited and underdeveloped, indicating a need for more efficient and scalable techniques.

From an application standpoint, there is a scarcity of high-value-added PFP-derived products with well-defined health benefits in the market, coupled with an underdeveloped supply chain. Addressing these issues—by deepening the mechanistic understanding, improving extraction technologies, and developing viable products and supply chains—will be a focal point for future research. Overcoming these challenges is essential to fully unlock the application potential and added value of PFP in the food industry.

## 8. Conclusions

PFP is characterized by a high content of bioactive compounds, including pectin, polyphenols, dietary fiber, and essential minerals. The enhanced utilization of PFP has the potential to contribute significantly to the reduction of agricultural waste and environmental pollution. This paper has reviewed recent advancements in the extraction of active components from PFP, their documented biological effects, and their emerging applications in the food sector. Evidence indicates that PFP serves as a valuable source of nutrients and bioactives for future diets, with the extracted components demonstrating various beneficial properties for human health, such as antioxidant, anti-diabetic, anti-cancer, anti-inflammatory, and antibacterial effects. Consequently, the utilization of PFP holds considerable promise within the food industry, with potential applications in functional beverages, nutritional products, plant-based meat alternatives, and as a source of natural pigments. In summary, PFP is regarded as a promising functional food raw material due to the multifunctional properties inherent in its bioactive components.

## Data Availability

No new data were created or analyzed in this study.

## Abbreviations

The following abbreviations are used in this manuscript:

Abbreviation	Full Term
PFP	Passion fruit peel
DF	Dietary Fiber
PPFP	Purple Passion Fruit Peel
VA	Vitamin A
YPFP	Yellow Passion Fruit Peel
VC	Vitamin C
P	Phosphorus
S	Sulfur
Na	Sodium
Mg	Magnesium
K	Potassium
Zn	Zinc
Ca	Calcium
HMP	High-Methoxy Pectin
LMP	Low-Methoxy Pectin
HG	High Galacturonic Acid Polysaccharides
RGI	Rhamnogalacturonic Acid Polysaccharide I
RGII	Rhamnogalacturonic Acid Polysaccharide II
XG	Xylogalacturonic Acid Polysaccharides
TPC	Total Phenolics
TFC	Total Flavonoid
NADES	Natural Deep Eutectic Solvents
UAE	Ultrasound-assisted Extraction
TTC	Total Terpenoids
UA	Ursolic Acid
dw	Dry Weight
GAE	Gallic Acid Equivalent
C3G	Cyanidin-3-Glucoside
EFP	Enzymatically PFP
OEA	Anti-Inflammatory-Related Metabolites
SDF	Soluble Dietary Fiber
GABA	Gamma-Aminobutyric Acid
CP	Citrus Pectin
SIC	Synbiotic Ice Cream
